# Concurrent administration of heparin and activated protein C in a patient with pulmonary embolism and severe sepsis with positive outcome

**DOI:** 10.4103/0972-5229.58544

**Published:** 2009

**Authors:** Deven Juneja, S. Mohan, Vivek V. Veturi, Palepu B. Gopal

**Affiliations:** **From:** Department of Anesthesia and Critical Care Medicine, Global Hospital, Lakdi-ka-pul, Hyderabad, India

**Keywords:** Activated protein C, heparin, pulmonary embolism, severe sepsis

## Abstract

Results of the PROWESS trial suggested that heparin may reduce the efficacy of recombinant human activated protein C (rhAPC) and the XPRESS study also showed increased bleeding complications in patients receiving heparin with rhAPC. Although it has been shown that heparin prophylaxis may be used along with rhAPC, no study has shown the interaction between continuous heparin infusion and rhAPC. Here, we report a case of severe sepsis with pulmonary embolism who was concurrently administered heparin and rhAPC infusions, with positive results and no bleeding complications.

## Introduction

Severe sepsis (SS) is the commonest cause of mortality in the intensive care.[1] Several biological products with anticoagulant activity have been tested in clinical trials as complementary treatment for septic patients. However, recombinant human activated protein C (rhAPC) is the only one which has shown to reduce mortality in SS and has been recommended in patients at high risk of death and no absolute contraindication related to bleeding risk or relative contraindication that outweighs the potential benefit.[2–5] The phase 3 trial assessing the efficacy of rhAPC, the PROWESS Trial,[2] had excluded the patients who required anticoagulation for a documented or suspected deep-vein thrombosis (DVT) or pulmonary embolism (PE) due to increased risk of bleeding complications. It also raised the possibility that heparin might reduce the efficacy of rhAPC as the risk reduction due to rhAPC was less in the group of patients exposed to heparin. A further trial, the XPRESS study,[6] showed that heparin for DVT prophylaxis does not reduce the efficacy of rhAPC, and hence it should be continued when rhAPC is started in the event of the patient developing sepsis. No study has shown the effect of continuous heparin infusion on rhAPC or vice versa. Here, we report a case of SS with pulmonary embolism who was concurrently administered heparin and rhAPC infusions with positive results.

## Case Report

A 49-year-old hypertensive, non-diabetic male patient who was a known case of bilateral osteoarthritis of the knees, was admitted for bilateral total knee replacement. The patient was on Atenolol 100 mg and Ecosprin 50 mg preoperatively.

Surgery was uneventful, and he was transferred to a surgery ward after one day observation in the surgical intensive care unit (ICU) with an opioid epidural analgesia. DVT prophylaxis with low molecular weight heparin (LMWH) was started after surgery. On the third postoperative day, he developed sudden onset of breathlessness and dizziness associated with fever, hypotension and oxygen desaturation. However, there was no tachypnea, and the lungs were clear on auscultation. Dopamine infusion was started, and epidural opioids were stopped when the patient remained hypotensive even after fluid challenge; the patient was subsequently shifted to the ICU.

**Figure 1:**
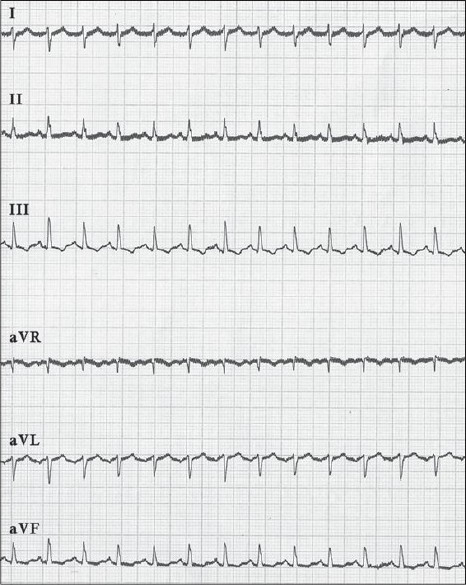
Electrocardiograph showing S wave in lead I, Q wave in lead III and T wave inversion in lead III (S_1_Q_3_T_3_ pattern)

**Figure 2:**
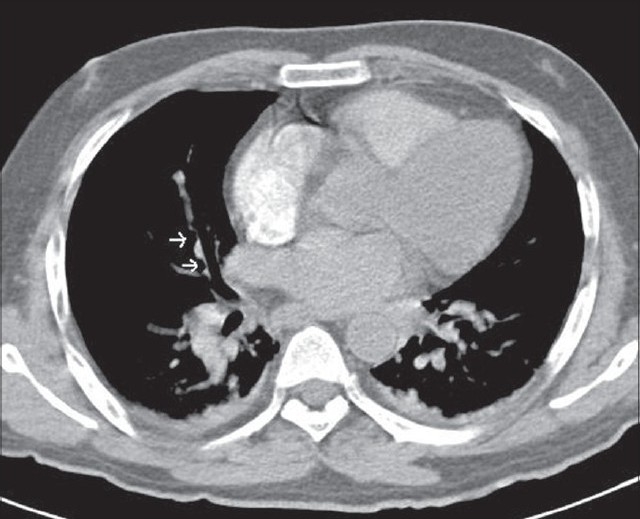
CT pulmonary angiography scan showing the filling defects (marked by arrows) in the right lower lobe segmental artery, which is suggestive of pulmonary embolism

In the ICU, a central line was inserted, central venous pressure was recorded (13-14 mm Hg), nor-adrenaline was started and dopamine was stopped. Chest X-ray was normal, and arterial blood gases revealed a pH of 7.382, PaO2 of 94 mmHg (12.5 kPa), PaCO2 of 19 mmHg (2.5 kPa), and base deficit of 14 mEq/L on 10 L/min of oxygen through face mask. Electrocardiography showed *S1Q3T3* pattern [Figure 1] and 2D Echo revealed plethoric inferior vena cava with mildly dilated right atrium and ventricle. D-Dimer was highly positive, but bilateral leg vein Doppler was negative for DVT. Because of high probability of PE, a CT pulmonary angiogram was performed, which revealed filling defects in the right lower lobe segmental artery with bilateral pleural effusion [Figure 2].

Patient was immediately started on heparin infusion at 1000 IU/h (14 IU/kg/h). Laboratory values are given in Table 1.

**Table 1:** Test parameters of the patient along with heparin, activated protein C and continuous renal replacement therapy support

Parameter of interest	Pre-op	1^st^ PO day	3^rd^ PO day	4^th^ PO day	5^th^ PO day	6^th^ PO day	7^th^ PO day	8^th^ PO day	9^th^ PO day	10^th^ PO day
APTT, s	32	36	120	94.7	115.7	102	91.3	56	54	61
Platelets, × 10^11^/L	2.47	1.37	2.08	1.79	1.45	1.05	0.97	0.84	0.37	0.27
INR	1	1.23	9	1.5	1.66	1.99	1.99	1.34	1.36	1.45
TLC, per mm^3^	7700	4900	11200	7800	16600	17100	12200	12200	11600	12100
Creatinine, mg/dl	0.9	1.3	2.7	2.1	2.2	2.2	1.4	1.4	1.4	3.4
Heparin infusion	−	−	+	+	+	+	+	+	+	+
APC	−	−	−	−	+	+	+	+	−	−
CRRT	−	−	−	−	+	+	+	+	−	−

PO - Postoperative; APTT - Activated partial thromboplastin time; INR - International normalized ratio, TLC - Total leukocyte count; APC - Activated protein C; and CRRT - Continuous renal replacement therapy

The patient continued to deteriorate with progressive hypotension, increased need for vasopressors and oxygen support, with reduced urine output. Heparin was increased to 1200 IU/h (17 IU/kg/h). Synovial fluid culture from knees grew MRSA, and hence, the antibiotics were changed from ciprofloxacin to piperacillin with tazobactam and linezolid in view of associated severe sepsis although blood cultures were negative.

On the fifth postoperative day, patient had to be intubated and put on mechanical ventilation due to worsening hypoxia on non-invasive ventilation. The epidural catheter was removed. For progressive hypotension, dopamine, dobutamine and vasopressin were sequentially added along with a low dose steroid. He became anuric and hence was started on continuous renal replacement therapy (CRRT). In view of SS with multi-organ failure (APACHE II score - 26), rhAPC was started (24 mcg/kg/h) and heparin infusion was continued. The dose of heparin was adjusted according to activated partial thromboplastin time (APTT) levels even if it meant increasing the dose beyond 15 units/kg/h, which is generally recommended as the maximum limit for a patient on rhAPC (Xigris information booklet).

Forty-eight hours after starting rhAPC, the patient started to show gradual improvement in oxygenation and blood pressure. Vasopressors were slowly tapered off over the next 3 days. There were no signs of bleeding anywhere or significant thrombocytopenia while heparin and rhAPC infusions were continued. On the eighth postoperative day, the patient was extubated onto noninvasive ventilatory support.

On the ninth postoperative day, CRRT was stopped as the patient started to produce urine. rhAPC was stopped after 96 h infusion.

On the 11^th^ postoperative day, a repeat CT pulmonary angiogram was performed which showed complete resolution of the embolus. Hence, heparin was stopped and the patient was maintained on LMWH and oral anticoagulation- (acitrom - acenocoumarol). Intermittent dialysis was also discontinued as the patient's renal parameters improved and his urine output was adequate. The patient was subsequently transferred to the surgery ward. His further hospital stay was uneventful, and he was discharged from the hospital on the 15^th^ postoperative day.

## Discussion

Septic patients are at increased risk of venous thromboembolic events, which may be due to advanced age, chronic cardiopulmonary disease, recent surgery, immobilization, or in-dwelling vascular catheters. Even though the concurrent use of heparin and APC is not contraindicated in such patients and heparin prophylaxis has been recommended even if a patient is on rhAPC, rhAPC has to be used with caution and the heparin dose should be less than 15 IU/kg/h (Xigris information booklet). To the best of our knowledge, no other case has been reported with concurrent use of heparin infusion and rhAPC.

Sepsis is characterized by a systemic inflammatory and procoagulant response to infection. The rationale behind anticoagulant treatments is that certain factors such as APC, antithrombin (AT) and Tissue Factor Pathway Inhibitor (TFPI) are depleted, and the use of recombinant technology may replenish them.[2–5] Although heparin does not replenish what sepsis patients have depleted, it binds to and activates AT which reduces thrombin generation and fibrin formation, which may help in combating sepsis. Heparin also has many immunomodulatory actions that may affect the systemic response to sepsis.[7] It has been shown in a small cohort study that the early administration of intravenous therapeutic dose of unfractionated heparin may be associated with decreased mortality when administered to patients with septic shock, especially in patients with higher severity of illness.[4]

Earlier data supported the possibility of heparin reducing the efficacy of rhAPC.[2] It was suggested that the activation of protein C *in vivo* can be blocked by administration of low levels of heparin as heparin leads to the inhibition of thrombin, which is vital for the activation of protein C. Moreover, APC has its own unique inhibitor, the APC inhibitor, which is stimulated by relatively high levels of heparin (5-10 μ/ml).[8]

Recently, heparin and rhAPC have shown significant anticoagulant synergy in plasma due to decreased thrombin generation by the prothrombinase complex.[9] Further, the reduction in sepsis-induced interactions between isolated platelets, neutrophils, and endothelial cells that is caused by rhAPC may be attenuated by low-dose heparin, which may play a role in preserving microvascular patency in patients with septic shock.[10] Emergent data from *in vitro* studies[11] have also suggested that patients with SS might benefit from a treatment with combinations of anticoagulant agents that might have worked to control sepsis in our patient too.

In addition to heparin, which might have been beneficial in controlling sepsis, rhAPC also might have helped in the treatment of PE in our patient as it has been shown to cause systemic anticoagulation and enhance fibrinolysis by inhibiting plasminogen activator inhibitor activity. rhAPC has also been used in managing acute myocardial infarction patients after thrombolysis.[12]

We did not observe any bleeding complications inspite of giving more than the recommended dose of heparin (up to 17 IU/kg/h) along with rhAPC in our patient, which may be due to the close monitoring of coagulation parameters. Therefore, it seems prudent that rhAPC and heparin can be given concurrently, with positive results, in selected patient groups under close monitoring without any increased risk of bleeding. Moreover, it seems that there may be some cross benefit of using heparin and rhAPC in septic patients with thrombotic complications.
